# Incorporating Psychological Science Into Policy Making: The Case of Misinformation

**DOI:** 10.1027/1016-9040/a000493

**Published:** 2023-07-14

**Authors:** Anastasia Kozyreva, Laura Smillie, Stephan Lewandowsky

**Affiliations:** 1 Center for Adaptive Rationality, Max Planck Institute for Human Development, Berlin, Germany; 2 Joint Research Center, European Commission, Brussels, Belgium; 3 School of Psychological Science, University of Bristol, UK; 4 School of Psychological Sciences, University of Western Australia, Australia; 5 Department of Psychology, University of Potsdam, Germany

**Keywords:** misinformation, disinformation, harmful content, regulation, policy making

## Abstract

The spread of false and misleading information in online social networks is a global problem in need of urgent solutions. It is also a policy problem because misinformation can harm both the public and democracies. To address the spread of misinformation, policymakers require a successful interface between science and policy, as well as a range of evidence-based solutions that respect fundamental rights while efficiently mitigating the harms of misinformation online. In this article, we discuss how regulatory and nonregulatory instruments can be informed by scientific research and used to reach EU policy objectives. First, we consider what it means to approach misinformation as a policy problem. We then outline four building blocks for cooperation between scientists and policymakers who wish to address the problem of misinformation: understanding the misinformation problem, understanding the psychological drivers and public perceptions of misinformation, finding evidence-based solutions, and co-developing appropriate policy measures. Finally, through the lens of psychological science, we examine policy instruments that have been proposed in the EU, focusing on the strengthened Code of Practice on Disinformation 2022.

## Misinformation as a Policy Problem

Misinformation is a global problem in need of urgent solutions, primarily because it can encourage people to adopt false beliefs and take ill-informed action – for instance, in matters of public health (e.g., Loomba et al., 2021; Schmid et al., 2023; Tran et al., 2020). Although democratic societies can sustain some amount of unverified rumors or outright lies, there is a point at which willfully constructed alternative facts and narratives can undermine the public’s shared reality and erode trust in democratic institutions (Lewandowsky, Smillie, et al., 2020). Democracy requires a body of common political knowledge in order to enable societal coordination (Farrell & Schneier, 2018). For example, the public must be aware that the electoral system is fair and that an electoral defeat does not rule out future wins. Without that common knowledge, democracy is at risk. The seditious attempts to nullify the 2020 US election results have brought that risk into sharp focus (Jacobson, 2021).

Although not a new issue per se, misinformation has become a pressing global problem due to the rising popularity of digital media. Indeed, people around the world see the spread of false information online as a major threat, ranked just behind climate change (Poushter et al., 2022). Misinformation is therefore both a research problem – spanning the fields of network science, social sciences, and psychology – and a policy problem, defined as “a disconnection between a desired state and the current state of affairs” (Hoornbeek & Peters, 2017, p. 369).

The misinformation problem can be approached in a variety of ways, ranging from media literacy campaigns and government task forces to laws penalizing the act of sharing fake news (for an overview of actions taken across 52 countries, see Funke & Flamini, n.d.; see also Marsden et al., 2020). Worryingly, “fake news” laws in authoritarian states (e.g., Russia) are used to control the public conversation, effectively stifling opposition and freedom of the press (The Economist, 2021).

In the EU, when misinformation does not constitute outright illegal speech (e.g., consumer scams, incitement to terrorism) but is rather “legal but harmful,” choosing appropriate policy instruments is largely a matter of balancing the threat of harmful falsehoods against fundamental human rights and societal interests. This balancing act calls for evidence and expertise, which in turn require a successful interface between science and policy, including knowledge brokerage that facilitates a dialogue between the two distinct communities (Gluckman et al., 2021; Topp et al., 2018).

## How Can Scientific Research Inform Misinformation Policy?

**Figure 1:**
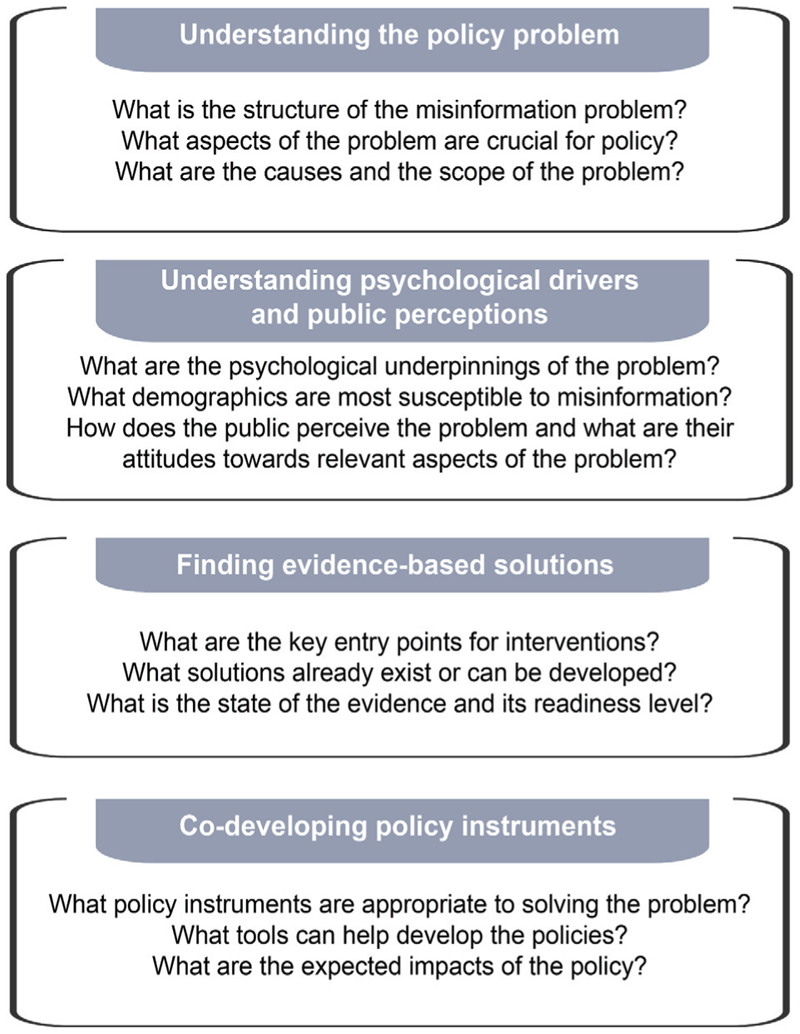
Building blocks of science-based policy for the case of misinformation.

In this article, we discuss how misinformation policy can be informed by scientific research. We outline four building blocks for evidence-informed misinformation policy – understanding the policy problem, understanding psychological drivers and public perceptions, finding evidence-based solutions, and co-developing policy measures – then focus on how a specific piece of the EU co-regulatory framework, the strengthened Code of Practice on Disinformation 2022 (“the Code”; European Commission, 2022a), draws on findings and arguments from cognitive science (Figure 1).

### Understanding the Policy Problem

To address a policy problem, a thorough understanding of the problem in question is crucial. Policies to manage the misinformation problem should take into account an understanding of the types of false and misleading content, the harms misinformation might cause, its distribution in online networks, and the factors that contribute to its spread.

#### Defining Misinformation and What It Means for Policy

“Misinformation” is often used by the research community and the general public as an umbrella term for various types of false or misleading content, including “fake news” (entirely false claims masquerading as news), unconfirmed rumors, half-truths, and factually inaccurate or misleading claims, conspiracy theories, organized disinformation campaigns, and state-sponsored propaganda. Misinformation can be defined along several dimensions, including degree of inaccuracy, presence of malicious intent, harmful outcomes, and risk of harmful outcomes (see, e.g., Kozyreva et al., 2020; Wardle & Derakhshan, 2017).

Determining the degree of inaccuracy of misinformation is a crucial first step. Online platforms are not in a position to independently establish whether information posted by their users is factual; they therefore generally consider accuracy only to the extent that it can be evaluated by external experts (e.g., Twitter, n.d.).^1^ Platforms outsource judgments on accuracy to external and certified fact-checking organizations. The credibility and expertise of organizations that correct misinformation are important to ensure the successful reduction of misperceptions (Lewandowsky, Cook, Ecker, Albarracín, et al., 2020; Vraga & Bode, 2017). Crucially, fact-checking organizations rarely work with binary definitions of truth; rather, they apply rating scales to both content and its sources. For instance, PolitiFact uses a 6-point rating scale to determine truthfulness (Drobnic Holan, 2018); Meta’s scale allows fact-checkers to classify content as false, altered, partly false, missing context, satire, or true (Meta, 2022); and News-Guard assesses websites based on nine journalistic criteria, then assigns an overall rating on a 100-point scale of trustworthiness (e.g., Aslett et al., 2022). Unlike binary approaches, rating scales can paint a more nuanced picture of misleading content online.

Misleading content can also be classified according to the intent behind sharing it. For instance, Wardle and Derakhshan (2017) distinguished three types of “information disorders”: *misinformation* (false or misleading content created and initially shared without malicious intent), *disinformation* (false, fabricated, or manipulated content shared with intent to deceive or cause harm), and *malinformation* (genuine information shared with the intent to cause harm, – e.g., hate speech and leaks of private information). Although this classification establishes some useful general distinctions, in practice it is often impossible to differentiate between misinformation and disinformation because the intent is difficult to infer. Furthermore, if misinformation and disinformation differ only in intent and not in content, they can have the same psychological effects on an individual, and their consequences can be equally harmful. We argue that policies that address misinformation should define the intent using measurable characteristics. For instance, policies could focus on the behavioral proxies of intent, such as signs of coordinated inauthentic behavior (e.g., fake accounts) and repeated sharing of falsehoods.

Another crucial dimension in defining misinformation is the severity of harm it can cause or has already caused. From the policy angle, false and misleading information is considered harmful when it undermines people’s ability to make informed choices and when it leads to adverse consequences such as threats to public health or to the legitimacy of an election (European Commission, 2020a). Research must therefore establish the specific aspects of online misinformation that threaten individuals and society and thus warrant policy attention. It should also examine how misinformation contributes to other events (e.g., elections, measures to contain pandemics) and how it affects people’s behavior and relevant antecedents of behavior (e.g., intentions, attitudes; see Schmid et al., 2023). For example, relative to factual information, exposure to misinformation can reduce people’s intention to get vaccinated against COVID-19 by more than 6 percentage points (Loomba et al., 2021). Misinformation about climate change undermines people’s confidence in the scientific consensus (van der Linden et al., 2017), and exposure to climate misinformation reduces people’s acceptance of the science more than accurate information can increase it (Rode et al., 2021).

Paying specific attention to harmful misinformation is particularly important in light of the proportionality principle. The severity of harm (represented, e.g., by the number of casualties or other adverse consequences) is one of the most impactful factors in people’s decisions to impose limits on online speech (Kozyreva et al., 2023) and hate speech (Rasmussen, 2022). Online platforms’ policies have – at least until recently – largely reflected that point. For example, Twitter’s approach to misinformation explicitly evoked the proportionality principle, claiming that actions against misinformation should be proportionate to the level of potential harm and whether the misinformation constitutes a repeated offense (Twitter, n.d.). However, the fate of such policies at Twitter and other platforms remains uncertain, highlighting the need for transparent and consistent rules for content moderation, independent of the whims of individuals with vested interests.

Risk and uncertainty posed by misinformation are also crucial for policy making. *Risk* refers to the likelihood of harmful consequences actually occurring, and *uncertainty*, in this context, refers to variance in the estimates of risk. Even small risks can warrant policy attention, especially if their potential consequences could be highly damaging. Moreover, the higher the uncertainty associated with estimates of potential harm, the more policy attention the issue might deserve. For example, greater uncertainty about climate change implies a greater probability of adverse consequences and therefore a stronger, rather than weaker, need for mitigating measures (Lewandowsky, Risbey, Smithson, & Newell, 2014; Lewandowsky, Risbey, Smithson, Newell, et al., 2014).

#### What Are the Causes and the Scope of the Problem?

Understanding the policy problem involves investigating underlying causes and the conditions that facilitate the spread of misinformation, as well as establishing the scope of the problem in a measurable way. Establishing causality is crucial for addressing the root of the misinformation problem. For instance, if social media were found to increase people’s exposure to harmful misinformation and the associated change in behaviors, it would be legitimate to expect that a change in social media might influence social well-being. In the absence of causality, this expectation does not hold. Overall, there is sufficient evidence to suggest that misinformation has causal effects on people’s behavior and attitudes (Bursztyn et al., 2020; Loomba et al., 2021; Rode et al., 2021; Simonov et al., 2020). At the same time, the nature of these causal effects is ambiguous and dependent on cultural context. For instance, the positive effects of digital media are intertwined with serious threats to democracy, and these effects are distributed differently between established and emerging democracies (Lorenz-Spreen et al., 2022).

Efforts that contribute to understanding the scope of the problem include monitoring misinformation across platforms, tracing the problem’s origins to specific actors (e.g., political actors, foreign interference, superspreaders), and investigating how features of online environments can amplify misinformation and influence people’s behavior. Identifying the motivations of those who intentionally spread falsehoods and curbing incentive structures that facilitate the spread of misinformation are also important for controlling the sources of the problem. For instance, Global Disinformation Index staff (2019) found that online ad spending on disinformation domains amounted to $235 million a year. The Code and the Digital Services Act (DSA) of the European Union, therefore, have several provisions aimed at demonetizing such content.

Monitoring programs that track misinformation across platforms are crucial; under the DSA they are an obligation for most major platforms (European Commission, 2020b). Independent organizations also contribute to the task (e.g., the Virality Project and the EU’s COVID-19 monitoring and reporting program both monitor pandemic-related disinformation). Independent monitoring makes it possible to estimate the size of a threat and to establish which platforms and sources are hotspots for misleading content. Most studies estimate that political misinformation (or “fake news”) online constitutes anywhere from 0.15% (Allen et al., 2020) to 6% (Guess et al., 2019) of people’s news diet, but there are indicators of considerable cross-platform variation. For example, Altay and colleagues (2022) found that in 2020, “generally untrustworthy news outlets, as rated by NewsGuard, accounted for 2.28% of the web traffic to news outlets and 13.97% of the Facebook engagement with news outlets” (p. 9). Similarly, Bradshaw and Howard (2019) showed that from 2017 to 2019, the number of countries with disinformation campaigns more than doubled (from 28 to 70) and that Facebook remains the main platform for those campaigns. These findings are particularly concerning since Facebook is the most popular social media platform for news both globally and in Europe (Newman et al., 2022).

Finally, an important factor in tracing the spread of misinformation and implementing measures to moderate it is detection. As the pressure to detect problematic content proactively and at scale mounts, platforms are increasingly relying on algorithmic tools (Gorwa et al., 2020) for detecting misinformation, moderating content, and attaching warning labels. However, these tools are fraught with problems, such as a lack of transparency and the inevitable occurrence of false positives, when acceptable content is removed, and false negatives, when posts violate platform policies but escape detection (for an informed overview and discussion of the topic, see Gorwa et al., 2020).

#### Implications for Policy

Identifying key characteristics of misinformation and the aspects of misinformation that merit policy attention is especially important for defining policy objectives. Making policy decisions about truth is a notoriously difficult task, not only because people may disagree about what constitutes truth, but also because limiting speech can pose dangers to democracy (for a discussion, see Sunstein, 2021). Because the majority of false and misleading information is not classified as illegal content in the EU and its member states, EU policy measures addressing misinformation focus primarily on harmful but legal content, including bots, fake accounts, and false and misleading information that could lead to adverse consequences and societal risks. Risk and harm feature prominently in policy objectives. For instance, the DSA requires online platforms whose number of users exceeds 10% of the EU population to assess systemic risks such as risks to public health and electoral processes. The intent behind sharing misinformation is also for selecting appropriate measures on the policy level. For example, in EU policy, when misinformation is spread without intent to deceive, it “can be addressed through well-targeted rebuttals and myth busting and media literacy initiatives,” whereas malicious disinformation “needs to be addressed through other means, including actions taken by governments” (European Commission, 2020a, p. 4).

Major challenges for policy include consistency, transparency, and cross-platform integration. The Code, therefore, requires its signatories to commit to developing common definitions or “a cross-service understanding of manipulative behaviors, actors and practices not permitted on their services” (European Commission, 2022a, Commitment 14, pp. 15–17) and to dedicate transparency centers and task forces to these issues.

## Understanding Psychological Drivers and Public Perceptions

Policies to manage the misinformation problem should also take into account the cognitive and behavioral factors involved in exposure to and perception of misinformation. This requires a clear picture of how people interact with online platforms and what makes them particularly susceptible to misinformation, as well as which groups of people are most vulnerable.

### What Are the Psychological Underpinnings of the Problem?

There are various psychological drivers of belief in misinformation (for an overview, see Ecker et al., 2022; Zmigrod et al., 2023; for an account on what makes people succumb to science denial, see Jylhä et al., 2023). For instance, people tend to accept information as true by default. Although this default makes sense given that most of an individual’s daily interactions are with honest people, it can be readily exploited. The perceived truthfulness of a message increases with variables such as repetition; motivated cognition, which can be triggered by information that is congruent with one’s political views; and failure to engage in deliberation that could have revealed that the information was unsubstantiated (i.e., inattention-based approach; see Pennycook & Rand, 2021).

Moreover, misinformation may press several psychological hot buttons (Kozyreva et al., 2020). One is negative emotions and how people express them online. For instance, Vosoughi and colleagues (2018) found that false stories that went viral were likely to inspire fear, disgust, and surprise; true stories that went viral, in contrast, triggered anticipation, sadness, joy, and trust. The ability of false news to spark negative emotions may give it an edge in the competition for human attention; moreover, digital media may encourage the expression of negative emotions like moral outrage (Crockett, 2017). In general, people are more likely to share messages featuring moral–emotional language (Brady et al., 2017). Because misinformation is not tied to factual constraints, it can be designed to trigger attentional and emotional biases that facilitate its spread.

Another factor in the dissemination of false and misleading information is the business models behind social media, which rely on immediate gratification, engagement, and attention. These goals determine the design of the algorithms that customize social media news feeds and the recommender systems that suggest content. Although not in themselves malicious, algorithmic filtering and personalization are designed to amplify the most engaging content – which is often sensational or negative news, outrage-provoking videos, or conspiracy theories and misinformation (Lewandowsky & Pomerantsev, 2022). For example, Mozilla’s (2021) study confirmed that YouTube actively recommended videos that violate its own policies on political and medical misinformation, hate speech, and inappropriate content. Problems associated with the amplification of harmful content might emerge not merely due to psychological biases and predispositions, but, crucially, because technology is designed to exploit these weaknesses. For example, the structural properties of social networks may be enough in themselves to cause challenges such as echo chambers, regardless of how rational and unbiased its users are (e.g., Madsen et al, 2018).

### What Demographics Are Most Susceptible to Misinformation?

Misinformation generally makes up a small fraction of the average person’s media diet, but some demographics are disproportionately susceptible (Allen et al., 2020; Grinberg et al., 2019; Guess et al., 2019). Strong conservatism, right-wing populism, and advanced age are predictors of increased engagement with misleading content (van der Linden et al., 2020). Converging evidence across studies in several countries indicates that the propensity to believe in COVID-19 conspiracy narratives is linked to right-wing voting intentions and conservative ideologies (Leuker et al., 2022; Roozenbeek et al., 2020). A recent cross-cultural study found that supporters of political parties that are judged as extreme on either end of the political spectrum (extreme left-wing and especially extreme right-wing) have a higher conspiracy mentality (Imhoff et al., 2022).

### How Does the Public Perceive the Problem and What Are the Public Attitudes to the Relevant Aspects of the Problem?

People’s perceived exposure to misinformation online is high: In an EU study, 51% of respondents using the internet indicated that they had been exposed to misinformation online and 37% stated that they had been exposed to “content where you could not easily determine whether it was a political advertisement or not” (Directorate-General Communication, 2021, p. 61). In a recent global survey, 54% of respondents were concerned about the veracity of online news, 49% said they had come across misinformation about COVID-19 in the last week, and 44% had encountered misinformation about politics in the last week (Newman et al., 2022). Furthermore, 70% of respondents across 19 countries see the spread of false information online as a “major threat” (Poushter et al., 2022). Although perceived exposure to misinformation may differ from actual exposure, a perceived prevalence of misinformation online can suffice to increase mistrust in media and political institutions (e.g., CIGI-Ipsos, 2019).

Given that the spread of misinformation is inextricably entangled with platforms’ algorithms, public attitudes towards algorithms and data usage are highly relevant to policymakers. Social media news feeds, viewing suggestions, and online advertising have created highly personalized environments governed by nontransparent algorithms, and users have little control over how the information they see is curated. A recent survey showed that most respondents in Germany (61%) and Great Britain (61%) and approximately half in the United States (51%) deem personalized political advertising unacceptable (Kozyreva et al., 2021). In all three countries, people also objected to the use of most personal data and sensitive information that could be collected for personalization. Nearly half (46%) of EU citizens worry about the use of personal data and information by companies or public administrations (European Commission, 2021d), and only 25% of people globally trust social media to use their data responsibly (Newman et al, 2022).

### Implications for Policy

An understanding of the psychological drivers behind misinformation and public perceptions of false and misleading information is particularly relevant for regulations on platform design. Platforms are currently not free of design that might exploit human psychology for profit, for instance, using persuasive choice architecture or information to capture people’s attention (Kozyreva et al., 2020). A policy must also take into account the cognitive implications of online technologies and protect the public against potential manipulation. Researchers have argued that protecting citizens from manipulation and misinformation and protecting democracy requires a redesign of the online architecture that has misaligned the interests of platforms and consumers (Lewandowsky & Kozyreva, 2022; Lewandowsky & Pomerantsev, 2022; Lewandowsky, Smillie, et al., 2020). It is crucial to restore the signals that make informed decision-making possible (Lorenz-Spreen et al., 2020) and to offer users more control over their data and the information they are shown. It is important to note that understanding the psychology of misinformation does not always produce an actionable policy agenda. For example, the finding that extreme conservative ideology is predictive of conspiracy mentality (van der Linden et al., 2020) cannot inform impartial policies.

In order to address the imbalance between online platforms and the public and to increase transparency, the DSA introduced “wide-ranging transparency measures around content moderation and advertising” (European Commission, 2021b, p. 2). These measures include increased algorithmic accountability, in particular with regard to how information is prioritized and targeted. For instance, the DSA gives users more control over recommender systems and the power to refuse targeted advertising; it also bans targeted advertising to vulnerable groups. To minimize the risks associated with online advertising, profiling and microtargeting practices must be transparent and purveyors of disinformation must be barred from purchasing advertising space. Political advertising and microtargeting practices are also addressed in the proposal for political advertising legislation (European Commission, 2021c), which aims to provide a unifying framework for political ads online and offline.

The strengthened Code also encourages its signatories to commit to safe design practices by facilitating “user access to tools and information to assess the trustworthiness of information sources, such as indicators of trustworthiness” (European Commission, 2022a, p. 23) and increasing the accountability of recommender systems (e.g., by “prohibiting, downranking, or not recommending harmful false or misleading information, adapted to the severity of the impacts and with due regard to freedom of expression and information”; p. 20).

## Finding Evidence-Based Solutions

Psychological science can also provide evidence for interventions and solutions aimed at reducing the spread of misinformation.

### What Are the Key Entry Points for Interventions?

Kozyreva et al. (2020) identified four types of entry point for policy interventions in the digital world: regulatory (e.g., legislative initiatives, policy guidelines), technological (e.g., platforms’ detection of harmful content and inauthentic behavior), educational (e.g., school curricula for digital information literacy), and socio-psychological (e.g., behavioral interventions to improve people’s ability to detect misinformation or slow the process of sharing it). Entry points can inform each other; for instance, an understanding of psychological processes can contribute to the design of interventions for any entry point, and regulatory solutions can directly constrain and inform the design of technological and educational agendas.

### What Solutions Already Exist or Can Be Developed?

Misinformation research offers many ways of slowing the spread of dangerous falsehoods and improving people’s ability to identify unreliable information (see Kozyreva, Lorenz-Spreen et al., 2022; Roozenbeek et al., 2023). There are behavioral and cognitive interventions that aim to fight misinformation by debunking false claims (Ecker et al., 2022; Lewandowsky, Cook, Ecker, Albarracín, et al., 2020), boosting people’s competencies through digital media literacy (Guess et al., 2020) and lateral reading (Breakstone et al., 2021; Wineburg et al., 2022), inoculating people against manipulation (Basol et al., 2020; Cook et al., 2017; Lewandowsky & van der Linden, 2021; Roozenbeek & van der Linden, 2019), and implementing design choices that slow the process of sharing misinformation – for instance, by highlighting the importance of accuracy (Pennycook, Epstein, et al., 2021) or introducing friction (Fazio, 2020). These tools and interventions stem from different disciplines, including cognitive science (Ecker et al., 2022; Pennycook & Rand, 2021), political and social psychology (Brady et al. 2017; Van Bavel et al., 2021), computational social science (Lazer et al., 2018), and education research (Caulfield, 2017). They also rely on different conceptual approaches (e.g., nudging, inoculation, boosting, techno-cognition – for an overview see, Kozyreva et al., 2020; Lorenz-Spreen et al., 2020) and different research methodologies to test their effectiveness (e.g., Pennycook, Binnendyk, et al., 2021; Wineburg et al., 2022). Although interventions may differ in terms of scalability, field studies have shown that accuracy prompts (Pennycook & Rand, 2021) and psychological inoculation campaigns on social media are effective at improving misinformation resilience at scale (Roozenbeek et al., 2022).

New challenges in online environments call for new competencies in information management. This might require going beyond what has traditionally been taught in schools. For instance, to efficiently navigate online information, people must be able to ignore large amounts of it and focus on that which is relevant to their goals (Kozyreva, Wineburg, et al., 2022). They must also be able to evaluate scientific information themselves (Osborne et al., 2022).

### What Is the State of the Evidence?

Not all evidence is created equal. Research on behavioral interventions should only be applied to a policy if the quality of evidence and its readiness level is appropriate (IJzerman et al., 2020). For instance, some studies are run on nonrepresentative samples and might not be generalizable enough to serve a policy’s objectives. It is therefore important to consider the generalizability and replicability of empirical studies. Methodological steps such as metascience and research synthesis for policy-relevant problems and solutions can help mitigate these challenges (Topp et al., 2018). Different types of research syntheses might be required to address the misinformation problem, including conceptual overviews and reports, systematic reviews, meta-analyses, expert reviews (Lewandowsky, Cook, & Ecker, 2020), and living reviews (Elliott et al., 2021).

### Implications for Policy

Evidence-based solutions are especially important for policies geared toward empowering citizens to deal with misinformation. Current EU policies foster digital media and information literacy through design choices and educational interventions; for example, improving media and information literacy through educational interventions is one of the priorities of the new Digital Education Action Plan (2021–2027), a policy initiative directed in part at helping EU citizens develop digital skills and competences.

The strengthened Code encourages platforms to highlight reliable information of public interest (e.g., information on COVID-19) and prioritize reliable content from authoritative sources (e.g., with information panels, banners, pop-ups, maps, or prompts) in order to empower users. It also focuses on enabling users to flag harmful false or misleading information and on introducing warnings that content has been identified as false or misleading by trusted third-party fact-checkers.

Research on interventions against misinformation has advanced considerably in recent years. In practical terms, this means that there is a toolbox of interventions that can be applied to the challenges of online environments (Kozyreva, Lorenz-Spreen, et al., 2022). In addition, platforms themselves have been implementing and testing various interventions on a large scale (e.g., Twitter Safety, 2022). However, internal reports on the effectiveness of platform interventions rarely go beyond reporting mere percentages and are not transparent methodologically. It is therefore particularly important that platforms heed the Code’s call for data sharing and cooperation with researchers (see also Pasquetto et al., 2020).

Finally, interventions aimed at fostering media literacy and empowering online users should not be regarded as a substitute for developing and implementing systemic and infrastructural solutions (see Chater & Loewenstein, 2022). Instead, as the “Swiss cheese model” for mitigating misinformation suggests (Bode & Vraga, 2021), different measures and interventions should be implemented as multiple lines of defense against misinformation.

## Co-Developing Appropriate Policy Instruments

The final building block of developing science-based policies to tackle the misinformation problem is developing evidence-informed policy instruments.

### What Policy Instruments Are Appropriate?

In the Better Regulation Toolkit (European Commission, 2021a), “evidence” refers to data, information, and knowledge from multiple sources, including quantitative data (e.g., statistics and measurements), qualitative data (e.g., opinions, stakeholder input), conclusions of evaluations, and scientific and expert advice. The toolkit makes no specific reference to psychological science. However, contemporary policy-making techniques such as stakeholder and citizen dialogue can identify knowledge gaps and different ways of framing a problem, and psychological science lends itself well to these techniques (e.g., strategic foresight, citizen engagement, and workshops to establish the values and identities triggered by a policy area; Lewandowsky, Smillie, et al., 2020; Scharfbillig et al., 2021). Research in psychological science can thus help identify evidence that is critical for establishing the appropriate type of policy instrument.

### What Tools Can Help Develop the Policies?

Several tools can assist with policy development. One tool is the foresight exercise, which is particularly suitable when policymakers face systemic challenges that require them to address multiple issues simultaneously while facing high uncertainty. In a foresight exercise, policymakers and key stakeholders imagine possible futures that may arise in response to their actions, without specifying links between policy decisions and outcomes. Creating, exploring, and examining radically different possible futures can help policymakers uncover evolving trends and dynamics that may have a significant impact on the situation at hand. For instance, Lewandowsky, Smillie, et al. (2020) explored four possible futures of the European online environment: The “struggle for information supremacy” scenario, which assumes that the European information space will be marked by high degrees of conflict and economic concentration; the “resilient disorder” scenario, in which the EU has fostered a competitive, dynamic, and decentralized information space with strong international interdependence, but faces threats from disinformation campaigns; the “global cutting edge” scenario, which foresees a world in which societal and geopolitical conflict have been reduced significantly, while high degrees of competition and innovation have led to the emergence of a dynamic, global information space; and the “harmonic divergence” scenario, which assumes that regulatory differences and economic protectionism between nations have resulted in a fractured global information space. From these scenarios, participants in a stakeholder workshop generated five classes of potential drivers of change that might determine the future (society, technology, environment, economy, and policy). This process highlighted the two most important drivers, which were also the most uncertain: the changing economic paradigm and conflicts and cyberattacks. Identifying those drivers is an important step toward future action by policymakers: At the very least, it suggests that resources should be allocated to further study of those drivers with a view towards reducing uncertainty.

### What Are the Expected Impacts of the Policy?

Any regulation proposal needs to address potential impacts and the outcomes of the policy for relevant actors and society as a whole. In the EU, this is achieved through impact assessment, a standard methodology adopted by the EU Commission to address the potential social, economic, and environmental impacts of a policy (Adelle & Weiland, 2012; European Commission, n.d.). An impact assessment comprises a structured analysis of policy problems and corresponding policy responses. It involves developing policy objectives and policy options, as well as ascertaining the options’ subsidiarity, proportionality, and impact. The assessment also considers effective procedures for monitoring and evaluation. Impact assessments provide the evidence base for a range of policy options and may result in a preferred option.

## EU Policy Approaches to Misinformation: The Strengthened Code of Practice on Disinformation

In liberal democracies such as the EU, online platforms are currently the primary regulators of speech on the internet, thus leaving the power to make and enforce rules in the hands of unelected individuals at profit-driven companies. To address this issue, the EU Commission has developed an array of policies combining stricter regulations for platform design and co-regulation guidelines for misinformation and harmful content (European Commission, 2022b; see also Helberger, 2020; Marsden et al., 2020). Both regulatory and self-regulatory policy instruments for addressing misinformation are currently under development (see Table E1 in the Electronic Supplementary Material, ESM 1, for an overview).

The DSA, the centerpiece of EU policy measures, establishes the EU-wide regulatory framework for providers of digital services. It also designates a special category of service providers – Very Large Online Platforms (VLOPs) – under the assumption that the risk of harm from the dissemination of misinformation is connected to the size and reach of the platform (Broughton Micova, 2021). VLOPs are required to manage systemic risks related to the dissemination of illegal content, potentially negative effects on fundamental rights, and intentional manipulation of their services, including “any actual or foreseeable negative effects in relation to gender-based violence, the protection of public health and minors and serious negative consequences to the person’s physical and mental well-being.” (European Parliament, 2022, p. 75).^2^

The self-regulatory Code is a central piece of EU policy on harmful misinformation (European Commission, 2022a). In force since October 2018, its signatories include major online platforms such as Meta, Google, and Twitter. A strengthened version of the Code was released in 2022, with 34 signatories agreeing to 44 commitments and 128 implementable measures. It features measures designed to strengthen platforms’ approach to misinformation, including more robust commitments to defund organized disinformation, limit manipulative behaviors, increase transparency in political advertising, and empower users, researchers, and fact-checkers. The strengthened Code also has a provision to improve cross-platform policy making.

In Table 1, we summarize the Code’s commitments, their significance from the perspective of cognitive science, and the VLOP signatories. As the Table shows, the user-empowerment section of the Code is the strongest from the perspective of cognitive science. For instance, psychological science research provides a variety of interventions to tackle the spread of misinformation (see also section “Finding Evidence-Based Solutions”), and insists on the importance of choice architectures that enhance user autonomy and facilitate informed decision-making (e.g., including trustworthiness indicators). However, several VLOPs have declined to sign up for some of the commitments in the user empowerment section (e.g., commitments 20 and 22). The importance of individual empowerment, as highlighted by cognitive science, appears to be a weakness for VLOPs. This will be especially significant if the Code evolves into a co-regulation instrument for the VLOPs under the DSA. Signatories of the EU Code published their first 6-monthly monitoring report on February 9, 2023 (https://disinfocode.eu/reports-archive/?years=2023). There are clearly discrepancies in the quality of reporting, particularly on the part of the platforms. It is clear that further development is needed to establish meaningful metrics for impact. Nevertheless, this is an important first step in increasing transparency and sharing insight and data from a broad stakeholder group.

## Conclusion

Former Google executives once called the internet and its applications “the world’s largest ungoverned space” (Schmidt & Cohen, 2013, p. 3). Meaningful legislation that protects citizens and institutions online must incorporate relevant evidence accessible and in a timely manner. However, many policymakers access evidence through procurement service providers, which traditionally do not provide insights from psychological science. Civil servants could therefore consider systematically including access to evidence from psychological science in their procurement contracts.

The regulatory and co-regulatory efforts in the EU are a milestone in creating online spaces that protect democracy and people’s best interests. Nevertheless, many problems and questions remain. First, achieving a balance of rights and interests while controlling the spread of misinformation is a difficult task. Some policy choices represent genuine dilemmas – for instance, pitting freedom of expression against the need to mitigate the harms of misinformation (Douek, 2021; Kozyreva et al., 2023). Second, policy making in a rapidly evolving online environment requires flexibility and constant updating. By the time legislation is published it might already be out of date. Third, platforms must be willing to cooperate in good faith. This has proven challenging so far, at least in part because the interests of platforms and the public are not always aligned (Lewandowsky & Kozyreva, 2022) and many large platforms do not provide researchers with adequate access to data (Pasquetto et al., 2020). To move forward, platforms, regulators, and researchers need to find a way to cooperate productively and act in a timely manner.

## Supplementary Material

Table E1

## Figures and Tables

**Table 1:** Cognitive foundations and signatory commitments under the strengthened Code of Practice on Disinformation 2022^1^

Commitment	Summary	Significance from the perspective of cognitive science	Very Large Online Platform (VLOP) adopters	VLOP non-adopters^2^
Scrutiny of ad placements (3 commitments)				
1. Demonitization	Disconnect ad revenues from disinformation, with independent audits	Monetary incentives can be powerful motivators of behavior. Global Disinformation Index staff (2019) estimate that a quarter billion dollars’ worth of advertising globally goes to sites flagged as disseminating disinformation.	Google (Ads) Meta (Facebook, Instagram) Microsoft (Ads) = all but 1.4 (*does not buy advertising*) Microsoft (LinkedIn) TikTok Twitter	Google (Search, YouTube) Meta (WhatsApp, Messenger) Microsoft (Bing)
2. Ads containing disinformation	Disrupt algorithmic amplification of disinformation	Algorithms prioritize engagement and can inadvertently amplify attention-grabbing content. Compared to verifiable information, misinformation is more emotional and negative (Carrasco-Farré, 2022) and more likely to inspire fear, disgust, and surprise (Vosoughi et al., 2018).	Google (Ads) Meta (Facebook, Instagram) Microsoft (Ads, LinkedIn) TikTok Twitter	Google (Search, YouTube) Meta (WhatsApp, Messenger) Microsoft (Bing)
3. Cooperation	Cooperation with fact-checkers	Fact-checking can reduce people’s beliefs in false information, especially when detailed refutations are provided. Source credibility and expertise also matter for successful corrections (Lewandowsky, Cook, Ecker, Albarracín, et al., 2020)	Google (Ads) Meta (Facebook, Instagram) Microsoft (Ads, LinkedIn) TikTok Twitter	Google (Search, YouTube) Meta (WhatsApp, Messenger) Microsoft (Bing)
Political advertising (10 commitments)				
4. Common definition	Adopt a common definition of “political and issue advertising”	NA	Google (Ads) Meta (Facebook, Instagram) Microsoft (Ads, LinkedIn) TikTok Twitter	Google (Search, YouTube) Meta (WhatsApp, Messenger) Microsoft (Bing)
5. Consistent political ads	Apply a consistent approach across political and issue advertising	NA	Google (Ads) Meta (Facebook, Instagram) Microsoft (Ads, LinkedIn) TikTok Twitter	Google (Search, YouTube) Meta (WhatsApp, Messenger) Microsoft (Bing)
6. Efficient labeling	Transparent labeling of political ads (incl. developing best practices, improving visibility, participating in research)	Although users pay some attention to these disclosures, they often do not enhance users’ knowledge of who paid for a given ad (Binford et al., 2021). Labels and associated information should be easy to understand and should provide the signals that make informed decision-making possible (Lorenz-Spreen et al., 2020). Platforms must continue to improve labeling on political ads and collaborate with researchers.	Google (Ads) = all but 6.5 Meta (Facebook, Instagram) = all but 6.5 TikTok = all but 6.5 Twitter = all but 6.5 - *not messaging apps*	Google (Search, YouTube) Meta (Messenger) = all but 6.5 Meta (WhatsApp) Microsoft (Ads, LinkedIn, Bing - *do not allow political or issue-based advertising*)
7. Verification	Ensure identity of ad sponsor is known	Identifying the source of sponsored content can help people use strategies for verifying its credibility (e.g., lateral reading; Wineburg et al., 2022).	Meta (Facebook, Instagram) Google (Ads) Twitter TikTok	Google (Search, YouTube) Meta (WhatsApp, Messenger) Microsoft (Ads, LinkedIn) = all but 7.3 (relates to reporting ads that may violate respective policies) Microsoft (Bing) - *does not allow political or issue-based advertising*
8. User-facing transparency	Transparent information on political ads	Transparency is an essential element of democratic governance (Fung, 2013).	Meta (Facebook, Instagram) Google (Ads) Twitter TikTok	Google (Search, YouTube) Meta (WhatsApp, Messenger) Microsoft (Ads, LinkedIn, Bing - *does not allow political or issue-based advertising*)
9. Transparency of targeting	Identify targeting for ads	Customizing messages, including political ads, based on a receiver’s personal characteristics is known as microtargeting. In microtargeting, digital fingerprints are used to infer personal attributes such as religion, political affiliation, and sexual orientation (Hinds & Joinson, 2018; Kosinski et al., 2013). Microtargeted political ads can be used to undermine democratic discourse. Most people oppose microtargeting of certain content (e.g., political ads) and microtargeting based on certain attributes (e.g., political affiliation; Kozyreva et al., 2021).	Meta (Facebook, Instagram) Google (Ads) Twitter TikTok	Google (Search, YouTube) Meta (WhatsApp, Messenger) Microsoft (Ads, LinkedIn, Bing - *do not allow political or issue-based advertising*)
10. Repositories	Full historical record of ads and targeting	NA	Google (Ads) Meta (Facebook, Instagram) TikTok Twitter	Google (Search, YouTube) Meta (WhatsApp, Messenger) Microsoft (Ads, LinkedIn, Bing - *do not allow political or issue-based advertising*)
11. Application programming interfaces (APIs)	Provide researchers and public access to data for research	Researchers have identified ways for online platforms to contribute to the study of misinformation, specifying how “increased data access would enable researchers to perform studies on a broader scale, allow for improved characterization of misinformation in real-world contexts, and facilitate the testing of interventions to prevent the spread of misinformation.” (Pasquetto et al., 2020, p. 2).	Google (Ads) Meta (Facebook, Instagram) TikTok Twitter	Google (Search, YouTube) Meta (WhatsApp, Messenger) Microsoft (Ads, LinkedIn, Bing - *do not allow political or issue-based advertising*)
12. Civil society	Permit scrutiny of advertising during elections	NA	None	Google (Ads, Search, YouTube) Meta (Facebook, Instagram, WhatsApp, Messenger) Microsoft (Ads, LinkedIn, Bing) TikTok Twitter - *not civil society organizations*
13. Ongoing collaboration	Continue to monitor evolving risks	Online misinformation and manipulation are moving targets and require consistent monitoring and updating of measures.	Google (Ads) Meta (Facebook, Instagram) TikTok Twitter	Google (Search, YouTube) Meta (WhatsApp, Messenger) Microsoft (Ads, LinkedIn, Bing - *do not allow political or issue-based advertising*)
Integrity of services (3 commitments)				
14. Impermissible manipulative behaviors (common understanding)	Platforms should adopt and implement publicly available policies for impermissible manipulative behaviors, maintain list of manipulative strategies	Knowing common manipulative strategies can help in preemptively inoculating people against them (e.g., Lewandowsky & van der Linden, 2021; Roozenbeek et al., 2022)	Google (Search, YouTube) Meta (Facebook, Instagram) Microsoft (LinkedIn, Bing) TikTok Twitter	Meta (WhatsApp, Messenger) Google (Ads)
15. AI transparency	Algorithms used for detection and content moderation should be transparent and respect user rights; manipulative practices for AI systems are prohibited	Misinformation policies should be consistent across platforms. Process of establishing transparent and consistent rules for content moderation can be informed by public attitudes (e.g., Kozyreva et al., 2023; Rasmussen, 2022).	Google (Search, YouTube) Meta (Facebook, Instagram) Microsoft (LinkedIn, Bing) TikTok Twitter	Meta (WhatsApp, Messenger) Google (Ads)
16. Cooperation and transparency	Cross-platform integration of efforts	NA	Google (Search = all but 16.2 – *no platform migration*, YouTube) Meta (Facebook, Instagram) Microsoft (LinkedIn, Bing = all but 16.2) TikTok Twitter	Google (Ads) Meta (WhatsApp, Messenger) Microsoft (Ads)
Empowering users (9 commitments)				
17. Media literacy	Extend users’ media literacy	The literature on various interventions aimed to improve users’ media literacy is extensive (e.g., Guess et al., 2020; Kozyreva, Lorenz-Spreen et al., 2022; Roozenbeek et al., 2022; Wineburg et al., 2022).	Google (Search, YouTube) Meta (Facebook, Instagram) Microsoft (LinkedIn, Bing) TikTok Twitter	Google (Ads) Meta (WhatsApp, Messenger) Microsoft (Ads)
18. Safe design	Disrupt algorithmic amplification of disinformation and increase safety of design	Design of online architectures has an impact on people’s decisions and behavior. Interventions via choice architectures and cognitive design principles can contribute to safer and autonomy-promoting online environments (e.g., Lorenz-Spreen et al., 2020).	Google (YouTube, Search = all but 18.1 - *does not allow for viral propagation*) Meta (Facebook, Instagram) Microsoft (LinkedIn, Bing = all but 18.1) TikTok Twitter	Google (Ads) Meta (WhatsApp, Messenger) Microsoft (Ads)
19. Transparency of recommender systems	Transparent AI and recommender systems	NA	Google (Search, YouTube) Meta (Facebook, Instagram) Microsoft (LinkedIn, Bing) TikTok Twitter	Google (Ads) Meta (WhatsApp, Messenger) Microsoft (Ads)
20. Provenance tools to check authenticity and accuracy of content	Authentication tools	Interactive, customizable labels and warnings can empower users.	Microsoft (Bing, LinkedIn)	Google (Ads, Search, YouTube) Meta (Facebook, Instagram, WhatsApp, Messenger – *other tools exist*) Microsoft (Ads) TikTok Twitter – *will explore the feasibility of this measure*
21. Fact-checking and flagging tools for accuracy	Provide users with labels and warnings	Several recent studies have confirmed the efficacy of labels and warnings in the context of misinformation, especially when corrections contained an alternative explanation (Ecker et al., 2010) and when information was identified as true or false while it was presented, or immediately afterwards (Brashier et al., 2021). When only some false information is flagged as false and true information is not affirmed, this may *raise* the perceived truth of other false information that is not flagged (Pennycook et al., 2020). Efficient labeling requires further research, as textual disclosure labels may go unnoticed (compared to graphic labels) (Dobber et al., 2021).	Google (Search = all but 21.2, YouTube) Meta (Facebook, Instagram) Microsoft (LinkedIn, Bing = all but 21.2 – *do not allow for viral propagation*) TikTok Twitter	Google (Ads) Meta (WhatsApp, Messenger) Microsoft (Ads)
22. Indicators of trustworthiness of sources	Provide users with trustworthiness indicators and pointers to trustworthy sources	Trustworthiness of content can be measured with reasonable objectivity (e.g., by checking a site’s record of reporting accuracy). Professional fact-checkers rely extensively on exogenous cues of trustworthiness: They spend very little time on the site itself, instead searching other sites in a process called “lateral reading” (Wineburg & McGrew, 2019). Users rely mainly on endogenous cues when evaluating the trustworthiness of content, which is often ineffective. People can be taught to use exogenous cues and engage in lateral reading. Identifying these cues requires further research using platform data (to select trustworthiness indicators) and behavioral experimentation (to verify that they are useful).	None	Google (Ads) Meta (Facebook, Instagram, WhatsApp, Messenger – *other tools exist*) Microsoft (Ads) Google (Search, YouTube = all but 22.7) Microsoft (LinkedIn, Bing) = all but 22.1, 22.2, 22.3, and 22.7 TikTok = all but 22.7 – *measures are unnecessary* Twitter (all but 22.7)
23. Flagging functionalities	Allow users to flag content	Users can play an important role in correcting misinformation (Bode & Vraga, 2018; Pennycook & Rand, 2019)	Google (Search, YouTube) Meta (Facebook, Instagram) Microsoft (LinkedIn, Bing) TikTok Twitter	Google (Ads) Meta (WhatsApp, Messenger) Microsoft (Ads)
24. Transparent appeal mechanism	Permit transparent appeal when content is removed	NA	Google (YouTube) Meta (Facebook, Instagram) Microsoft (LinkedIn) TikTok Twitter	Google (Ads, Search) Meta (WhatsApp, Messenger) Microsoft (Ads) Microsoft (Bing) – *does not post user content*
25. Messaging apps	Introduce friction or labels to limit viral propagation	Introducing friction can slow down the spread of misinformation (e.g., Fazio, 2020). Although such prompts are used by several major social media companies (e.g., Twitter and Meta), evidence of its effectiveness for messenger apps is lacking.	Meta (WhatsApp, Messenger)	Google (Ads, Search, YouTube) Meta (Facebook, Instagram) Microsoft (Ads, LinkedIn, Bing) Tiktok Twitter - *not messaging apps*
Empowering the research community (4 commitments)				
26. Automated access to non-personal data	Provide researchers and public access to data for research	Researchers have identified ways for online platforms to contribute to the study of misinformation, specifying how “increased data access would enable researchers to perform studies on a broader scale, allow for improved characterization of misinformation in real-world contexts, and facilitate the testing of interventions to prevent the spread of misinformation.”	Google (Search = all but 26.2, YouTube) Meta (Facebook, Instagram) Microsoft (LinkedIn, Bing) TikTok Twitter	Google (Ads) Meta (WhatsApp, Messenger) Microsoft (Ads)
27. Governance structure for access to data	Create governance structure for access to data	(Pasquetto et al., 2020, p. 2).	Google (Search, YouTube) Meta (Facebook, Instagram) Microsoft (LinkedIn, Bing) TikTok Twitter	Google (Ads) Meta (WhatsApp, Messenger) Microsoft (Ads)
28. Cooperation	Cooperate with researchers		Google (Search, YouTube) Meta (Facebook, Instagram) Microsoft (LinkedIn, Bing) TikTok Twitter	Google (Ads) Meta (WhatsApp, Messenger) Microsoft (Ads)
29. Conducting research	Platforms continue research on disinformation and how to enhance public resilience against it	Platforms conduct internal research but rarely share their findings and data in a transparent way.	None	Google (Ads, Search, YouTube) Meta (Facebook, Instagram, WhatsApp, Messenger) Microsoft (Ads, LinkedIn, Bing) TikTok Twitter - *not research organizations*

*Notes*.

1 This table includes 29 out of 44 commitments. The excluded 15 commitments refer to measures aimed at empowering the fact-checking community and implementing the Code and are therefore less relevant for cognitive science. Signatories of the Code listed at: https://digital-strategy.ec.europa.eu/en/library/signatories-2022-strengthened-code-practice-disinformation.

2 Unless otherwise specified (in italics), reasons for not subscribing to commitments are either not indicated by the signatories of the Code or indicated to be not applicable.

## Footnotes

1 However, even in the presence of established scientific consensus, the issue of accuracy and who gets to decide what is true can be extremely divisive, especially when the topic is highly politicized and polarized – as is the case for many topics rife with misinformation (e.g., climate change denial, COVID-19 anti-vaccination).

2 Recital 106 suggests that rules on codes of conduct under this Regulation could serve as a basis for existing self-regulatory efforts at the EU level, including the meaning that VLOPs could become co-regulated under the DSA when the Code becomes a Code of Conduct.
