# Supplementary material for: Incorporating Psychological Science Into Policy Making: The Case of Misinformation
Source: Eur Psychol. Author manuscript; Available in PMC 2023 Nov 22. (PMC7615323; doi:10.1027/1016-9040/a000493)
Supplement: Table E1 [file EMS190996-supplement-Table_E1.pdf]

https://doi.org/10.1027/1016-9040/a000493

Electronic Supplementary Materials 1

Incorporating Psychological Science Into Policy Making: The Case of Misinformation

Table E1. Misinformation policy measures in the EU

Name	Description	Type of policy instrument & its status	Relevant policies/measures	Documents
Digital Services Act (DSA)	A legislative proposal by the European Commission that establishes a common set of rules, obligations, and accountability measures for providers of digital services aimed at ensuring a safe, accessible, predictable, and trusted online environment where fundamental rights are protected.	EU-level regulation.<br><br>Status: Awaiting formal adoption by the EU co-legislators	Key measures:<br>Prohibition of deceptive practices (e.g., dark patterns).<br>Mandating transparency in algorithmic targeting and advertising, including possibility to refuse targeting in advertising and disallowing targeting of vulnerable groups.<br>Mandating transparency and user control over parameters in recommender systems.<br>Risk assessment for very large online platforms.<br>External independent auditing.<br>Access to data and algorithms for vetted researchers.	European Commission, 2020: Proposal on a Single Market For Digital Services and Amending Directive<br>European Parliament, 2022: Amendments<br>Corrigendum<br>For an overview, see The DSA content
Code of Practice on Disinformation (The Code)	A self-regulatory tool launched in 2018, The Code underwent an initial assessment as part of the Commission's December 2020 European Democracy Action Plan.	Self-regulatory instrument.<br>Status: Assessing the progress - Signatories have six months for implementation.	Expected updates:<br>Reducing monetization of disinformation.<br>Improved approach to political ads, including labeling, transparency, and improved quality of repositories.<br>Comprehensive definition of manipulative and impermissible behaviors across platforms.<br>Strengthened commitments to limit impermissible	European Comission, 2018: Code of Practice on Disinformation<br>European Commission, 2021: European Commission Guidance on Strengthening the Code of Practice on Disinformation<br>European Commission, 2022: strengthened Code of Practice on

https://doi.org/10.1027/1016-9040/a000493

			manipulative behaviors.<br>Requirement to publish relevant policies according to certain standards.<br>Commitment to measures enhancing media literacy, including safe design and increased functionality for debunking and flagging and fact-checking.<br>Framework for access to data for research on disinformation.	Disinformation<br>European Commission, 2022: Signatories<br>For an overview, see the Agreement
Regulation on Political Advertising (RPA)	A regulation proposal that aims to regulate the European market of political advertising and proposes the rules that will apply to providers of political advertising services (both online and offline). This proposal is complementary to the DSA.	Regulation initiative.<br>Status: Feedback on commision adoption closed.	Obligation of transparency and record keeping.<br>Controlling targeting and amplification.<br>Tackling misleading content.	European Commission, 2021: Inception impact assessment<br>Proposal for a Regulation on the Transparency and Targeting of Political Advertising<br>Impact Assessment Report<br>Annexes<br>Opinion on Impact Assessment<br>For an overview including feedback see the political advertising initiative
European Democracy Action Plan (EDAP)	A set of policy actions to help European democracy face the challenges of the digital age, to empower citizens, and to build more resilient democracies across the EU. These actions include, among others, the DSA and the enhanced Code of Practice on Disinformation	Action plan.	Three pillars:<br>Promote free and fair elections.<br>Strengthen media freedom and pluralism.<br>Counter disinformation.	European Commission, 2020: Communication On the European Democracy Action Plan<br>For an overview see the press release
Digital Education Action Plan (2021–2027)	A renewed EU policy initiative to support the sustainable and effective adaptation of the education and training systems of	Action plan.	Two priority areas:<br>Fostering the development of a high-performing digital education ecosystem.	European Commission, 2018: Communication on Digital Education Action Plan<br>Commission Staff Working

https://doi.org/10.1027/1016-9040/a000493

	EU member states to the digital age.		Enhancing digital skills and competences for the digital transformation.	Document<br>European Commission, 2020: Commission Staff Working Document<br>Factsheet<br>Digital Education Action Plan 2021-2027<br>For an overview see the digital education initiative
European Declaration on Digital Rights and Principles for the Digital Decade	A declaration that aims to provide a clear reference point about the kind of digital transformation Europe promotes and defends. It states that the rights and freedoms enshrined in the EU's legal framework, and the European values expressed by the principles, should be respected online as they are offline.	Declaration.<br>Status: Awaiting endorsement by European Parliament and Council	Key principles:<br>Putting people at the center of the digital transformation.<br>Guaranteeing access to digital connectivity and to all key public services online across .<br>Promoting digital education and skills.<br>Protecting autonomy in interactions with algorithms and artificial intelligence systems.<br>Ensuring a safe, secure and fair online environment where fundamental rights are protected and responsibilities of platforms, especially large players and gatekeepers, are well defined.<br>Privacy and data protection by design.<br>Supporting the development and use of sustainable digital technologies.	European Commission, 2022: Communication: Establishing a European Declaration on Digital Rights and Principles for the Digital Decade<br>The Declaration on Digital Rights and Principles<br>The Staff Working Document<br>For an overview see the overview and press release
